# Hypoxic burden as a cause of cardiovascular morbidity in childhood obstructive sleep apnea

**DOI:** 10.1038/s41390-025-04153-3

**Published:** 2025-05-23

**Authors:** Plamen Bokov, Benjamin Dudoignon, Christophe Delclaux

**Affiliations:** Paris Cité University, AP-HP, Robert Debré Hospital, Department of Physiology and Sleep Laboratory, INSERM NeuroDiderot, Paris, France

## Abstract

**Background:**

To assess whether hypoxic burden (HB) is associated with cardiac autonomic nervous system dysfunction and increased blood pressure (BP) in otherwise healthy children with moderate to severe obstructive sleep apnea (OSA).

**Methods:**

Among 103 children with moderate-to-severe OSA, twenty pairs, matched for age, sex and obstructive apnea-hypopnea index (OAHI) were selected, with low (first quartile) or high (fourth quartile) HB in each pair: median [25th–75th percentiles]; age: 10.8 years [7.3; 13.2] vs. 11.4 [8.6; 13.5]; sex: 7 and 10 girls; z-score of body mass index: 1.50 [0.11; 2.43] vs. 2.40 [1.92; 2.70] (*p* = 0.012); OAHI: 8.6/hr [6.4; 13.3] vs. 11.1 [6.6; 17.2] and HB: 0.8%.min/h [0.3; 1.5] vs. 13.8 [10.1; 22.3], respectively.

**Results:**

Non-linear heart rate variability (HRV) indices obtained from polysomnography showed sympathetic overflow (Poincaré plot: decreased SD2) and weaker parasympathetic modulation (decreased SD1) in children with high versus low HB. The high versus low HB group had higher percentiles of office BP: systolic, 75th [61; 81] vs. 57th [47; 69], *p* = 0.049 and diastolic, 70th [60; 78] vs. 55th [46; 65], *p* = 0.007, adjusted for obesity and arousal index.

**Conclusions:**

Despite similar levels of OAHI, children with higher HB demonstrate parasympathetic withdrawal and increased daytime blood pressure.

**Impact:**

- The hypoxic burden predicts cardiovascular morbidity/mortality in adult obstructive sleep apnea syndrome.
- Our study shows that in children with moderate to severe obstructive sleep apnea, despite similar levels of obstructive apnea-hypopnea index, those with higher hypoxic burden show cardiac autonomic nervous system dysfunction and increased daytime blood pressure.
- This study is the first one suggesting that the hypoxic burden is a valuable marker of cardiovascular morbidity in childhood obstructive sleep apnea.

## Introduction

Sleep-disordered breathing ranges in severity from primary snoring to obstructive sleep apnea (OSA). In adults, sleep-disordered breathing is associated with adverse cardiovascular consequences, which are mediated, in part, by autonomic nervous system (ANS) dysfunction.^1^ Although sleep-disordered breathing is common in children, fewer pediatric studies have investigated these cardiovascular effects. A review found evidence of ANS dysfunction in children with sleep-disordered breathing during both wakefulness and sleep.^2^ This ANS dysfunction is a risk factor in childhood for subsequent young adult hypertension^3^ and consecutive morbidity.

It is thought that hypoxia dominant OSA, a subtype of OSA in which ventilatory changes during the night result in oxygen desaturation but not necessarily arousal from sleep, can increase sympathetic nervous system activity, and as a result may lead to an increased risk of cardiovascular disease.^4^ Although childhood OSA is associated with a lesser degree of intermittent hypoxia than adult OSA it deserves to be determined whether intermittent hypoxia per se leads to ANS dysfunction, independently of obstructive events in childhood.

To overcome the limitations of the apnea-hypopnea index (AHI), several studies have suggested characterizing nocturnal hypoxia in OSA, termed as hypoxic burden, which has a greater prognostic ability of cardiovascular disease events in adults than the AHI.^4^

The main objective of our retrospective case-control study was to assess whether intermittent hypoxia is associated with ANS dysfunction using heart rate variability indices in otherwise healthy children with moderate to severe OSA. This population was selected because it is less prone to spontaneous recovery^5^ and thus more exposed to long-term cardiovascular consequences. Since our main objective was to assess the intermittent hypoxia consequences, independently of obstructive AHI, children were matched for age, sex and obstructive AHI, while their hypoxic burden levels were different. The secondary objective was to evaluate the impact of hypoxic burden on blood pressure (BP) at the time of referral, as well as its effects on excessive daytime sleepiness and hyperactive behavior.

## Material and methods

### Selection of the patients

This cross-sectional case-control study complied with STROBE guidelines. From our database of 142 otherwise healthy children referred for treatment of moderate to severe OSA (obstructive AHI ≥ 5/h of sleep) and with hypoxic burden availability (*n* = 103), we selected pairs of children matched for age (±2 years), obstructive AHI ( ± 3 events), sex when possible and different levels of hypoxic burden. In a preliminary analysis of 500 otherwise healthy children referred for OSA suspicion, the median value of hypoxic burden was 1.3% min/hour in those without OSA and 5.1% min/hour in those with OSA (defined as OAHI > 2/h,^6^ *n* = 285). In this latter group with OSA, the 25th percentile of hypoxic burden was 2.0%  min/h, and the 75th percentile was 9.0% min/h. We thus matched one child belonging to the first quartile (<2.0% min/h) with one child of the fourth quartile (>9.0% min/h). Twenty pairs of children were matched since among our 103 patients with moderate to severe OSA only 20 children had a hypoxic burden <2.0% min/h, while 33 had a hypoxic burden >9.0% min/h.

### Overnight polysomnography

In-laboratory (children under 8 years old) or ambulatory (≥8 years) polysomnography studies were performed overnight using an Alice 6 LDx or PDX polysomnography system (Philips, Murrysville, PA) or Somté PSG system (Compumedics, Australia), all using the Nonin oximetry device. The following parameters were recorded: chest and abdominal wall motion using respiratory inductance plethysmography, heart rate by electrocardiogram, arterial oxygen saturation by pulse oximetry, airflow using a 3-pronged thermistor, nasal pressure by a pressure transducer, electroencephalographic leads (C3/A2, C4/A1, F3/A2, F4/A1, O1/A2, O2/A1), left and right electrooculograms, submental electromyogram, and tibial electromyogram. Experienced pediatric sleep physicians scored patients using standard pediatric sleep scoring criteria.^7^ Sleep stages were expressed as z-scores using the normative values of the polysomnography parameters of Scholle et al., as previously described.^8^

### Calculation of the hypoxic burden

**Fig. 1:**
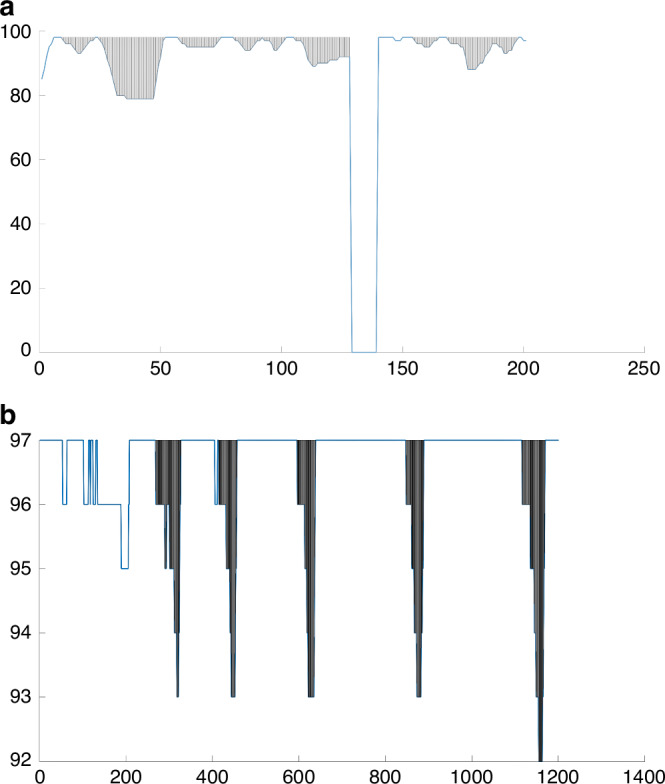
Representative examples of hypoxic burden calculation. **a** Example of hypoxic burden detection in the presence of abnormal SpO_2_ values (artifact with a sudden drop to 0%). The algorithm efficiently disregards these non-physiological values from the calculation of the hypoxic burden. Y-axis is SpO_2_; X-axis is time in s. **b** Example of hypoxic burden detection in a patient with slow and long lasting desaturations. Y-axis is SpO_2_; X-axis is time in s.

From polysomnography, the transcutaneous oxygen saturation (SpO_2_) signal was exported. The hypoxic burden was defined as the total area under the desaturation curve associated with a ≥ 3% desaturation event that was identified automatically. Prior to calculation of the total area under the desaturation curve, the SpO_2_ signal was pre-processed as follows. Periods of wake identified using manually scored sleep/wake states were discarded. Artefacts that indicated non-physiological values were also discarded (e.g., a decrease in SpO_2_ over two consecutive data points of more than 5%). A decrease in SpO_2_ by at least 3% for at least 4 s was deemed as a candidate event. For a robust area calculation (particularly for the events without SpO_2_ recovery to the baseline value), the subject-specific search window was obtained from an averaged desaturation curve. The average desaturation curve for each participant was determined by overlaying the SpO_2_ signal with respect to the end of events, as conducted by Azarbarzin et al.^9^ The desaturation areas bounded below by the SpO_2_ curve and above by a desaturation baseline that is based on the starting point of the desaturation were calculated. We did not use the pre-event baseline saturation defined as the maximum SpO_2_ during the 100 s prior to the end of the event that Azarbarzin et al. used^9^ because if an overshoot occurs during this period, this results in an over-correcting SpO_2_ from a previous desaturation, and a falsely elevated baseline would be chosen. The hypoxic burden was then obtained by adding these individual desaturation areas and dividing the total area by the sleep duration, with the units of hypoxic burden being % min/h. Figure 1 shows representative examples of hypoxic burden calculation.

### Heart rate variability analyses

Electrocardiographic recordings obtained during polysomnography were exported as EDF files. The files were then processed using HRV analysis software 1.1 downloaded at https://anslabtools.univ-st-etienne.fr and validated by Pichot et al.^10^

Four ECG periods over the polysomnography were selected, as previously described^11^:

1. A 5 min recording before sleep during wakefulness (30 min before sleep onset).
2. A 5 min recording during the first period of non-rapid eye movement (NREM) sleep (after the first 3 min).
3. A 5 min recording during the last rapid eye movement (REM) sleep period (after the first three minutes of the REM period).
  The 5 min ECG periods were selected as periods free of respiratory events, arousals and periodic limb movement. The selection of a specific epoch of sleep was justified based on the results of Kontos et al.^12^
4. The fourth period was selected that corresponded to the whole night.

A potential artefact identification and removal algorithm was applied to the entire RR interval data.

#### Linear analyses: 5 min and whole-night recordings

Time-domain variables included the mean sinus heart rate (HR), the standard deviation of the RR intervals (SDNN) and the root mean of squared successive differences (RMSSD, index of parasympathetic modulation).

#### Non-linear analyses on the whole-night recording

A Poincaré plot is graphed by plotting every RR interval against the prior interval, creating a scatter plot. We analyzed the Poincaré plot by fitting an ellipse to the plotted points. After fitting the ellipse, we derived two non-linear measurements, SD1 and SD2. The SD1 and SD2 indexes carry similar information to the spectral density power of the high frequency and low frequency bands but have the advantage of easier calculation and less stationarity dependence. SD1 measures short-term HRV, while SD2 measures short- and long-term HRV in ms. SD1 may provide information on the parasympathetic activity, whereas SD2 seems inversely related to sympathetic activity.^13^ SD1 was the primary outcome since we hypothesized that higher level of hypoxic burden would be associated with decreased parasympathetic modulation (see Sample size calculation).

Two other non-linear methods were used: the fractal dimension and the Lyapunov exponent.^14^ The higher the fractal dimension, the more irregular the signal is, i.e., the greater the dynamic complexity is. The algorithm proposed by Katz was used.^15^ The Lyapunov exponent is a measure of the system dependency on the initial conditions, but it also quantifies the predictability of the system.^14^ The value of the Lyapunov exponent increases, corresponding to lower predictability, as the degree of chaos becomes higher; a positive Lyapunov exponent is a strong indicator of chaos.

Sympathetic and parasympathetic tone refer to the steady-state level of sympathetic and parasympathetic nervous system activity that is maintained under resting conditions, while sympathetic and parasympathetic modulation refer to the oscillations in sympathetic and parasympathetic nervous system activities around the respective steady-state or mean level, quantified in the HRV metrics.

### Blood pressure measurements

BP was measured in the seated position and after 5 min rest on the morning of the hospital evaluation. Three measurements spaced 5 min apart were performed with an oscillometric device (Datascope Accutorr Plus™, Paramus, NJ) on the right arm, with appropriate pediatric cuff sizes. The means of the systolic and diastolic BP of the last two measurements were reported. Since normal BP varies during childhood with sex and age, or height, all BP measurements were expressed as percentiles. Percentiles for each BP measurement were extrapolated from Ln relationships that were established between BP norms and percentiles (50th, 90th and 95th percentiles) available from guidelines,^16^ as previously described.^11^

### Questionnaires

The modified Epworth Sleepiness Scale was used for the evaluation of excessive daytime sleepiness.^17^ Hyperactivity/inattention-related symptoms were evaluated using the Conners Abbreviated Symptom Questionnaire, which was completed by a parent.^18^ These questionnaires were selected since a recent study showed that the hypoxic burden is significantly associated with excessive daytime sleepiness^19^ and attention problems as an additional secondary objective.

### Statistical analyses

Sample size calculation: In a recent study describing the kinetics of arterial desaturation in 35 otherwise healthy children with moderate to severe OSA,^20^ their mean SD1 ± standard deviation value was 45 ± 25 ms. If the changes in HRV indices in OSA are related to intermittent hypoxia, one may hypothesize that children with OSA with a higher level of hypoxic burden would depict a blunted SD1 (decreased parasympathetic modulation) compared to their matched control children with lower hypoxic burden. To demonstrate a 20 ms difference of SD1 between the two groups (effect size), with a type I error rate of 0.05 and a type II error rate of 0.20 and a standard deviation of SD1 of 25 ms, we had to include at least 52 children (26 pairs). Finally, 20 pairs only were included, which was a potential limitation.

The results were expressed as median [25th–75th percentiles], since most indices did not follow normal distribution (HRV indices, for instance). Comparisons of the continuous variables between the two groups of children were performed using the Mann–Whitney U test. Categorical variables were compared using the chi-square test (Fisher’s exact test when necessary), and correlations were evaluated using the Spearman rank test. Multiple linear regression models used Box-Cox coefficients for transformations: Lyapunov -0.5; RMSSD, SD1, Fractal index of Katz 0.5; systolic, diastolic BP and arousal index were normally distributed. A *p*-value < 0.05 was deemed significant. The statistical analyses were performed with StatView 5.0 software (SAS Institute, Cary, NC) and with R software version 4.1.0 (multiple linear regression models and Box-Cox transformations). No correction for multiple testing was performed due to the pathophysiological design of the study.^21^

## Results

### Descriptive analyses

The median hypoxic burden of our population (*n* = 40) was 4.5%.min/h [0.8; 13.8]. The 20 matched pairs of children are described in Table 1, which shows that despite similar OAHI, all the indices characterizing intermittent hypoxia (ODI3%, SpO_2_ min and % time with SpO_2_ < 90%) were significantly different, while the arousal index was higher in the group with low hypoxic burden. The group with high hypoxic burden had higher z-score of body mass index (BMI). The sleep stages were not significantly different between the two groups of children.

**Table 1:** Characteristics of the two matched groups of otherwise healthy children with moderate to severe OSA.

Characteristics	Low hypoxic burden *N* = 20	High hypoxic burden *N* = 20	*P*-value	Adjusted *P*-value for obesity and arousal index
Age, years	10.8 [7.3; 13.2]	11.4 [8.6; 13.5]	0.561	
Sex, female/male, *n*	7 / 13	10 / 10	0.337	
Ethnicity, C/Af/As/M, *n*	4 / 11 / 4 / 1	9 / 8 / 1 / 2	0.210	
z-score of BMI	1.50 [0.11; 2.43]	2.40 [1.92; 2.70]	0.012	
obesity (BMI z-score > 1.645), *n*	10	18	0.014	
Questionnaires				
Epworth score	5 [3; 8]	8 [2; 11]	0.271	
Conners score	9 [3; 14]	9 [3; 12]	0.910	
Polysomnography				
OAHI/hour	8.6 [6.4; 13.3]	11.1 [6.6; 17.2]	0.317	
ODI/h	2.4 [1.4; 4.9]	9.7 [6.0; 17.8]	<0.001	
SpO_2_ min, %	92 [89; 93]	86 [82; 88]	<0.001	
Time SpO_2_ < 90%, % Total sleep time	0.0 [0.0; 0.0]	0.1 [0.0; 0.8]	0.006	
Mean desaturation duration, *s*	6 [5; 9]	8 [7; 10]	0.058	
Hypoxic burden, %.min/h	0.8 [0.3; 1.5]	13.8 [10.1; 22.3]		
Arousal index/hour	14.2 [12.5; 20.1]	8.8 [5.3; 15.0]	0.030	
z-score of N1	−0.51 [−1.24; 0.66]	−0.42 [−0.86; 0.36]	0.588	
z-score of N2	−0.24 [−1.09; 0.82]	0.06 [−0.92; 1.14]	0.402	
z-score of N3	0.76 [−0.76; 1.07]	0.04 [−0.49; 1.08]	0.725	
z-score of REM	−0.51 [−1.05; 1.08]	−0.14 [−1.33; 0.23]	0.607	
HRV indices				
*Wakefulness*				
RR, ms	631 [574; 708]	646 [613; 747]	0.661	
SDNN, ms	55 [41; 76]	52 [42; 65]	0.579	
RMSSD, ms	52 [45; 69]	34 [23; 55]	0.066	
*NREM sleep*				
RR, ms	736 [633; 879]	734 [656; 810]	0.745	
SDNN, ms	51 [30; 80]	38 [31; 57]	0.433	
RMSSD, ms	62 [36; 105]	43 [34; 50]	0.065	
*REM sleep*				
RR, ms	749 [675; 935]	730 [664; 821]	0.881	
SDNN, ms	103 [45; 113]	61 [47; 84]	0.086	
RMSSD, ms	71 [33; 101]	57 [42; 64]	0.194	
*Whole night*				
RR, ms	773 [681; 892]	728 [663; 828]	0.180	
SDNN, ms	114 [85; 141]	90 [73; 115]	0.123	
RMSSD, ms	74 [55; 100]	57 [44; 80]	0.042	0.036
SD1, ms	50 [41; 65]	40 [31; 51]	0.023	0.018
SD2, ms	146 [106; 185]	115 [96; 149]	0.168	
SD1/SD2 ratio	0.34 [0.28; 0.42]	0.35 [0.28; 0.38]	0.787	
Fractal index of Katz	1.87 [1.70; 2.02]	1.73 [1.62; 1.79]	0.020	0.007
Lyapunov	0.42 [0.34; 0.56]	0.34 [0.30; 0.42]	0.026	0.023
Daytime blood pressure (BP)				
Systolic BP percentile	57 [47; 69]	75 [61; 81]	0.041	0.049
Diastolic BP percentile	55 [46; 65]	70 [60; 78]	0.005	0.007

Ethnicities are Caucasian/African-Caribbean/Asian/Mixed.

### Primary outcome

A deficit in parasympathetic modulation was evidenced in the high hypoxic burden group when compared to the low hypoxic burden group during the whole night (lower RMSSD, lower SD1: primary outcome), with less irregular signal (lower fractal dimension) and higher predictability (lower Lyapunov exponent).

### Secondary outcomes

The children in the high hypoxic burden group had higher levels of both systolic and diastolic blood pressure compared to those in the low hypoxic burden group.

We did not observe differences of daytime sleepiness or attention problems between the two groups of children.

The correlations (Rho values) between the hypoxic burden and polysomnography parameters such as TST90, SpO_2_ minimal and ODI3% were 0.69 (*p* < 0.001), −0.65 (*p* < 0.001) and 0.65 (*p* < 0.001), respectively.

Since the z-score of BMI and arousal index were significantly different between the two groups of children, the last column of Table 1 provides the *p*-values adjusted for both obesity and arousal index (multiple linear regression models using Box-Cox coefficients for transformations: see Methods), showing that the adjustments did not alter the significance of our results.

## Discussion

The main results of our cross-sectional case-control study demonstrate that despite similar levels of respiratory events, children with higher intermittent hypoxia depict reduced heart rate variability mainly due to parasympathetic modulation withdrawal and increased blood pressure.

Our study design allowed us to control for the main bias constituted by age, sex and sleep apnea severity, which are important contributors of heart rate variability in children.^22–24^ Furthermore, our two groups of children were not statistically different in term of ethnicity, which is important since it also affects HRV indices.^25^ Overall, adequate matching of the two groups of children was an important prerequisite given the reduced sample size of our study. Nevertheless, the z-scores of BMI were significantly different, and the children with high hypoxic burden were more frequently obese, which may have influenced HRV indices.^26^

The median hypoxic burden of our population was very low compared to that observed in adult patients.^9^ Similarly, the other indices of desaturation (ODI, Time SpO_2_ < 90% and minimal SpO_2_) were also less severe compared to that observed in adult patients,^27^ which is consistent with the definition of OSA in children.^6^ A recent study of Walter et al. showed quite similar levels of hypoxic burden in typically developing children with moderate to severe OSA (median value: 6.2% min/h).^28^ We observed similar relationships with polysomnographic parameters in our population than those observed in adult patients with moderate to severe OSA.^9^ The higher arousal index in children with low hypoxic burden is related to the arousal-based scoring that recognizes hypopneas associated with electroencephalography-based arousals, with or without significant oxygen desaturation.^29^

Traditional markers of intermittent hypoxia (ODI, SpO_2_ min, TST90) show that intermittent hypoxia in childhood OSA is very limited. Severe desaturation inevitably occurs during a sequence of apnea, as elegantly demonstrated in adulthood OSA.^30^ Thus, the lower frequency of apnea in childhood OSA compared to in adulthood OSA explains the limited arterial desaturation and lower hypoxic burden. Nevertheless, the degree of SpO_2_ decrease and the duration of events in childhood OSA have been correlated with cerebral tissue oxygenation,^31^ which may have functional consequences such as excessive daytime sleepiness,^20^ even if we did not observe differences of daytime sleepiness or attention problems, as recently suggested.^32^

The hypoxic burden predicts cardiovascular disease mortality across adult populations.^9^ Such a demonstration in childhood is impossible, but surrogate risk factors of cardiovascular disease can be obtained. Despite the limited level of arterial desaturation in childhood, consequences are relevant. Liao et al. showed that sleep disorder breathing in healthy young children and in clinical patients is significantly associated with impaired cardiac autonomic modulation, i.e., sympathetic overflow and weaker parasympathetic modulation, which may contribute to an increased risk of acute cardiac events in persons with sleep disorder breathing, even before reaching the ‘high risk age’.^33^ We built on these previous results, showing that in children with moderate to severe OSA, those with a higher hypoxic burden related to OAHI show a defect in parasympathetic modulation in the whole night analyzes. Nevertheless, we show that in the different sleep stages, no significant differences were evidenced (only trends), the analyzes being made in periods free of any events, in contrast to the whole night analyzes. Thus, the modifications of the whole night analyzes are probably related to these events, namely arterial desaturations explaining that HRV has been successfully used to screen people for possible referral to a Sleep Laboratory.^23^ In the recent study of Walter et al., hypoxic burden was a negative predictor of sympathetic and parasympathetic activity in children with Down syndrome only, which could be related to the restricted number of typically developing children with moderate to severe OSA (*n* = 20) in their study.^28^ Our study strongly suggests that the link between OSA and cardiac autonomic nervous system dysfunction is related to hypoxic burden rather than to respiratory events, as in adults. Along the same lines, Van Eyck et al. showed that measures of hypoxia were related to increased sympathetic tone, suggesting that intermittent hypoxia is involved in autonomic dysfunction in overweight and obese children and adolescents.^34^

Finally, we show that the children with moderate to severe OSA and higher hypoxic burden had higher levels of blood pressure, which argues for already present cardiovascular consequences. Similarly, a recent meta-analysis showed that moderate to severe childhood OSA was associated with a risk of elevated systolic blood pressure in adulthood.^35^ The tracking of blood pressure levels from childhood to adulthood is well demonstrated, and higher BMI resulted in increasing blood pressure across trajectories, particularly for the higher blood pressure groups.^36^ The association between systolic blood pressure trajectories derived from childhood with subclinical cardiovascular risk in young adulthood has also been demonstrated.^37^ Thus, our children, mainly obese, with higher hypoxic burdens, are at risk of long-term cardiovascular disease, which has also recently been suggested by Chuang et al., who showed close relationships between intermittent hypoxia (ODI), overweight/obesity and hypertension.^38^ The independent effect of high hypoxic burden on the observed cardiovascular consequences is further demonstrated in our study (see Table 1). Autonomic dysfunction is present in obese children and adolescents, attributed to lower vagal activity.^39^ In adult patients with OSA, it has been shown that the patients in the hypoxic group were significantly younger and more obese, and their most common comorbidity was hypertension.^40^ Thus, it was important to demonstrate the independent effect of intermittent hypoxia, since repetitive arousals may also influence both HRV indices and blood pressure levels.^41,42^

Our cross-sectional descriptive study has inherent limitations. First, causality cannot be inferred from statistical associations. Nevertheless, the long-term cardiovascular consequences of cardiac autonomic dysfunction are well established, together with the beneficial effects of OSA treatment on hypoxic burden.^43^ Second, the calculation of the hypoxic burden in our study differed from the respiratory event-related hypoxic burden described by Azarbarzin et al.,^9^ since respiratory events were not taken into account. Nevertheless, we observed similar relationships with polysomnography parameters. Different methods of hypoxic burden calculation have been developed,^4^ and it remains to be evaluated whether one method provides benefits over the others. Third, given the small sample size of our study, the respective effects of obesity and high hypoxic burden on cardiovascular outcomes, excepting blood pressure, cannot be determined. In adult patients with OSA, higher hypoxic burden has been associated with an increased risk of cardiovascular disease and replacing BMI with other measures of visceral obesity in the fully adjusted model did not affect this association.^27^

Our study has clinical consequences. The cardiovascular consequences of childhood OSA remain debated.^6^ The definition of a cut-off value of hypoxic burden in childhood predicting long-term cardiovascular consequences is warranted. Our study was a first step toward this objective and is the first one suggesting that the hypoxic burden is a valuable marker from childhood.

In conclusion, our case-control study shows that despite similar levels of respiratory events, children with higher intermittent hypoxia and more obesity depict reduced heart rate variability mainly due to parasympathetic modulation withdrawal and increased daytime blood pressure.

## Data Availability

Data available on reasonable request to the corresponding author.
